# Physiological actions of “medical devices made of natural materials” (such as vegetal matrices) and non-pharmacological actions of “medical devices made of substances” (and as such artificial derivatives) as innovations introduced by Regulation 2017/745

**DOI:** 10.3389/fdsfr.2026.1807588

**Published:** 2026-04-13

**Authors:** Marcella Marletta

**Affiliations:** 1 Università Cattolica del Sacro Cuore, Rome, Italy; 2 Università Campus Biomedico, Rome, Italy; 3 Università Guglielmo Marconi, Rome, Italy

**Keywords:** matrix effect, mechanism of action, medical devices made of substances, natural materials, new approach, physiological state, substance, vegetal matrix

## Abstract

The European Union’s shift from Directive 93/42/EEC to Regulation 2017/745 changes how products made of chemically-defined substances and those made of natural materials should be conceived, evaluated, and regulated. In this study, it is argued that “medical devices made of substances” should be distinguished from “medical devices made of natural materials” (e.g., vegetal matrices) because the regulatory logic linked to “substances” is largely chemistry-centered and may be poorly suited to chemically variable systems whose performance, having the specific medical purpose of restoring a physiological state, emerges from supramolecular, network-level interactions. Regulation 2017/745 provisions addressing devices made of substances require well-defined compositions and identified constituents, consistent with reductionist approaches. In European regulatory practice, “substance” is commonly operationalized through well-defined molecular constituents as key players in pharmacological reasoning. In contrast, devices made of “natural materials” may modulate interconnected biofunctions and regulatory pathways in ways that cannot be reduced to a single constituent or to the sum of isolated constituents. This “matrix effect,” associated with redundancy and functional resilience, shifts quality/performance away from compositional markers and pharmacological, immunological or metabolic means toward a network interaction best framed as a physiological mode of action. Under Regulation 2017/745’s “New Approach,” manufacturers must justify applicable requirements, classification rules, and validation methods for the specific product. Although devices made of “substances” act via non-pharmacological, immunological, or metabolic means, “substance”-oriented requirements should not automatically be extended to natural-material devices whose quality and performance arise from whole-matrix properties. Clear differentiation will support proportionate regulation, innovation, and patient benefit.

## 1 Introduction

**FIGURE 1:**
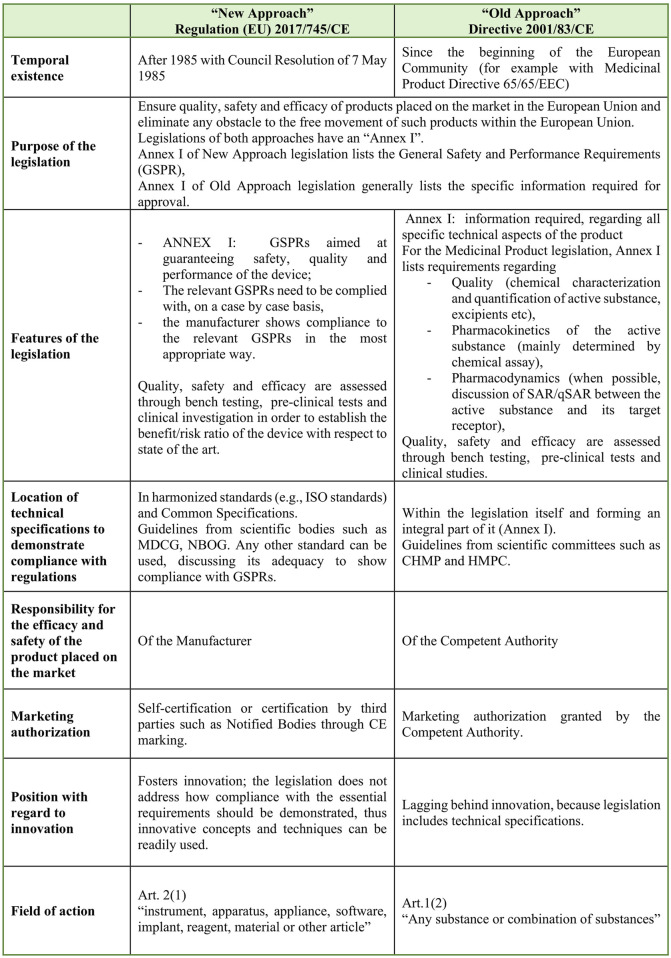
Main differences between the “New Approach” and the “Old Approach.” It can be observed that the Old Approach is product-oriented and lists the technical aspects to be complied with, while the New Approach gives less technical detail, thus being able to embrace a wider range of products. Abbreviations: CHMP, Committee for Human Medicinal Products; HMPC, Committee on Herbal Medicinal Products; GSPR, General Safety and Performance Requirements; MDCG, Medical Device Coordination Group; NBOG, Notified Bodies Operation Group; SAR/qSAR, structure–activity relationship/quantitative structure–activity relationship.

Medical Device Directive 93/42/EEC (MDD) (European Union, 1993) has guaranteed safe and effective devices to European physicians and patients for over 30 years. As a “New Approach Directive” (European Union, 1985; European Union, 2022), it included general and specific essential requirements that cover the device life cycle. General classification rules depended solely on invasiveness and time of contact with target tissues/organs. Features entailing specific hazards, such as animal derivatives or ancillary pharmacological substances, implied specific classification rules and certification requirements. The MDD definition of medical devices indicated a) device nature—instruments, software, or materials (translated in some languages such as Italian and German as “substance”); b) intended purpose; c) a non-pharmacological, immunological, metabolic (Ph.I.M.) mechanism of action (MOA). Essential requirements, classification, and certification were uniform for devices with similar invasiveness and duration of contact. In line with the New Approach, there were no obstacles to developing safe and effective devices made of substances or natural materials (Figure 1) and no need to distinguish between the two—whereas this distinction is necessary under Regulation (EU) 2017/745 (MDR) (European Union, 2017). The MDR introduces important innovations: it defines the roles and responsibilities of key economic operators (in particular the manufacturer), strengthens clinical evidence requirements, and broadens the definition of “medical device”. The nature of a device remains as in the MDD, except that “material” is consistently translated as “material” (and not “substance”). The medical purpose is broadened to include “prediction and prognosis of diseases” (first indent) and “modification of a pathological process and a physiological or pathological state” (third indent); a new fourth indent also specifies that a device may “…provide information by means of *in vitro* examination of specimens from the human body…”.

The concept of modifying a “state” is particularly relevant to medical devices made of natural materials (MDMNM), due to their “network over a network” mechanism accompanying physiological actions in a coordinated, circular, nonlinear manner and supporting the restoration of equilibrium (Marletta, 2024). Compared with the MDD, the MDR identifies a category of devices with risks related to toxicity and, where applicable, where systemic absorption was not adequately addressed by the previous framework. Introduced under Whereas 59 are specific requirements and a pharmacokinetically oriented classification rule for “…devices that are composed of substances or of combinations of substances that are absorbed or locally dispersed in the human body” (medical devices made of substances: MDMS). These provisions effectively delineate MDMS as composition-driven products and link them more closely to medicinal-product logic, tailored on the risks associated with artificial isolated molecules. Since constituents in nature are typically embedded within complex matrices rather than encountered as purified entities, distinguishing natural materials from MDMS is increasingly important to avoid constraining innovation. Clear interpretation of the available regulatory frameworks can support the development of safe and innovative therapeutic products aligned with “One Health” priorities, health technology assessment expectations, and clinical practice needs. This study analyzes the features of MDMS and MDMNM and summarizes emerging technical bases to assess the quality and mechanism of action of the latter.

## 2 Medical devices made of substances under Regulation 2017/745: reliance on Medicinal Product Directive Annex I and a constituent-based characterization framework

Regulation (EU) 2017/745 characterizes medical devices made of substances (MDMS) through dedicated requirements that, for specific endpoints, explicitly refer to the medicinal product framework. In particular, MDMS are subject to General Safety and Performance Requirement (GSPR) 12.2 and the related certification procedure in Annex IX, point 5.4(a), requiring the “…evaluation of absorption, distribution, metabolism, excretion (ADME), local tolerance, toxicity, interaction … (with other products)… and potential for adverse reactions.” Where applicable and not covered elsewhere by the MDR, these aspects should be evaluated in compliance with Annex I to Directive 2001/83/EC (Medicinal Product Directive, MPD) (European Union, 2001).

Annex I of the MPD reflects a reductionist (“Old Approach”) framework in which quality, efficacy, and safety are primarily grounded in chemical composition. Medicinal products are expected to be described as combinations of well-defined substances with an identifiable active principle, typically synthetic or purified from natural sources. Non-clinical testing in this framework is generally organized around defined constituents and their intrinsic risks in the human body. Where an “active molecule” is not readily evident—such as for preparations constituted of complex natural materials—Annex I-aligned approaches commonly operationalize assessment through the selection of markers to support quality evaluation and, where relevant, pharmacokinetic and pharmacodynamic considerations. Markers may include constituents known to contribute to therapeutic effect (active constituents/active markers); when such links are not established, analytical markers may be selected primarily to enable specification setting and batch control.

Thus, notwithstanding the broad definitions of “herbal substance” and “herbal preparation” in the MPD, Annex I Part III and associated EMA/HMPC quality guidelines require the selection of characteristic constituents/markers for quality control [e.g., (EMEA/HMPC/CHMP/CVMP/214869/2006, 2008; EMEA/HMPC/253629/2007, 2008; EMA/HMPC/186645/2008, 2010; EMA/HMPC/CHMP/CVMP/201116/2005rev3, 2022)], and although conceptually in HMPs, the herbal preparation in its entirety is regarded as a “complex multi-component system” “characteristic constituents need to be selected as specific for the herbal substance/preparation,” and quality specifications are typically anchored to the content (often within defined tolerances) of one or a few well-characterized constituents (EMA/HMPC/CHMP/CVMP/162241/2005rev3, 2022).

This is relevant when MDR assessment of MDMS draws on Annex I of MPD because it is most readily implemented when product identity and control can be expressed in terms of well-characterized constituents. From this regulatory-operational perspective, a “substance” is treated as matter with qualities (and key aspects of its evaluation) that can be represented and controlled through one or more well-defined molecular constituents or presented as such. Accordingly, where MDMS must refer to Annex I for endpoints such as ADME, assessment is most straightforward when the device can be described in terms of chemically well-defined substances (synthetic, purified from natural materials, or selected markers).

Other MDR provisions reflect the same chemically oriented framing. GSPR 23(2)(r) requires disclosure of the qualitative composition of the device and quantitative information on the main constituent or constituents responsible for achieving the principal intended action. Rule 21 classifies devices largely according to pharmacokinetic considerations, thus reinforcing Annex I settings, particularly where systemic absorption is required to achieve the intended purpose; in such cases, a medicinal product authority provides a scientific opinion on conformity with Annex I. In parallel, the REACH concept of UVCB substances accommodates complex, unknown, or variable composition while remaining rooted in a chemically oriented paradigm of “substances,” (ECHA, 2023). This overall approach is less readily applicable to materials with performance that cannot be represented or controlled through marker constituents, as is often the case for natural materials.

## 3 Supramolecular organization and the matrix effect in natural materials

Natural materials are governed by supramolecular, intermolecular, and intramolecular interactions among constituents, which modify the reactivity of individual components and give rise to the “matrix effect” (Wagner and Ulrich‐Merzenich, 2009). This is typical of living matter, which self-assembles and self-organizes to form supramolecular complex entities (Lehn, 2002) that show structural redundancy and functional resilience. Such organized complexity is central to biological systems (Whitesides and Grzybowski, 2002) and to their physiology (Xu et al., 2009; Bonnans et al., 2014). In this sense, the “native intelligence” of the material—the functional behavior that emerges from its native network of interactions—may be diminished when manufacturing entails extensive refinement or purification that strips constituents of their original interactions.

## 4 Experimental approaches for the characterization of substances and natural materials

In therapeutic settings, a product should be characterized according to the features that govern its therapeutic behavior. When qualitative–quantitative composition is tightly linked to effect, chemical assay of active constituents is the regulatory gold standard (Specifications and Control Tests on the Finished Product, 1992). This is typically the case for medicinal substances, the activity of which, consistently with their Ph.I.M. MOA, follows the structure–activity relationship (SAR) and quantitative SAR (qSAR) paradigm. However, when this link cannot be straightforwardly established, as with natural materials, methods able to capture the “matrix effect” are needed. The patent literature, which can provide examples of evolving approaches, includes Mercati (2025), who proposed a conceptual and experimental framework for validating natural matrices for therapeutic use on biological and biophysical bases as an alternative to validation through chemically definable substances obtained by isolation/synthesis or through traditional use (Mercati and Lucci, 2025a; Mercati and Lucci, 2025b; Mercati and Lucci, 2026). They argue that, despite intrinsic compositional variability, different batches of natural matrices can convey comparable biological effects. Accordingly, the activity of a natural matrix does not follow the SAR/qSAR paradigm, disconnecting biological output from strict qualitative–quantitative composition and suggesting that assay of a single marker is likely to be insufficient to predict effect. Approaches that capture supramolecular interactions among constituents are proposed as predictive of the matrix effect, enabling quality assessment of vegetal matrices, including batch-to-batch consistency. The continued development of such methods is laying the groundwork for a new state of the art anchored in matrix-level functional readouts.

## 5 Mechanism of action of substances versus natural materials: how the product interacts with the human body to reach the intended therapeutic effect

According to the FDA, “mode of action is the means by which a product achieves an intended therapeutic effect or action” (FDA, 2026a). The distinction between MOA and effect is important, since the same effect can be obtained by different MOAs. For example, heartburn may be alleviated by proton pump inhibitors through a pharmacological mode of action or by protecting the gastric mucosa through a chemical–physical MOA (Racchi et al., 2016).

MOA describes the paradigm through which a product interacts with the human body to achieve its intended effect. MPs and MDs differ not only in nature (substance vs. material), but also in their MOA, and nature and MOA are connected.

MPs are defined in Directive 2001/83 as having a pharmacological, immunological, or metabolic (Ph.I.M.) means, described through interactions at the molecular level and classically exemplified with ligand–receptor models, the effectiveness of which depends on a close relationship between the structure of the active substance and relevant biological targets—consistent with the SAR/qSAR paradigm (Racchi et al., 2016; Walsh and Schwartz-Bloom, 2004; European Commission, 2009)—and need to be present in adequate quantities (Infocuria, 2009). In this regard, MDCG 2022-5rev.1 (European Commission, 2024), aiming to add precision to MEDDEV 2.1/3rev3 definitions of these terms, explicitly introduces the term “substance” in the definitions of pharmacological, immunological, and metabolic means. The guideline states that “pharmacological means” is “…an interaction typically at a molecular level between a substance … and a constituent of the human body,” and provides examples including “interaction between a ligand (e.g., agonist, antagonist) and a receptor,” “interaction between a substance and membrane lipids,” and “interaction between a substance and components of the cytoskeleton.” The definitions of “immunological” and “metabolic” means are framed in the same way, where the achieved effect is due to the “action initiated by a substance” for the immunological means and the “action of a substance” for the metabolic means. Specifically, assigning these means to “substances”—well-defined molecules—clarifies from a regulatory standpoint that Ph.I.M. means involve an interaction between a “substance” and the human body. This does not imply that all substances act through Ph.I.M. means. For example, MDMS act through non-Ph.I.M. MOAs such as chemical and physical modes of action (Sardi et al., 2018). It does, however, suggest that materials that cannot be reconducted to being a “substance” do not fall within a Ph.I.M. MOA. This occurs with a natural matrix. As noted above, the observation that different batches, despite the intrinsic measurable differences in molecular composition, may consistently produce comparable biological effects suggests that its activity cannot be explained by classical SAR/qSAR models, involving instead network interactions of the matrix as a whole. In such cases, the MOA should be congruously described as non-Ph.I.M. Relevant patent literature again includes Mercati (2025), who argues that when the natural material network interaction modulates multiple connected and coordinated functions, producing a change in the overall biological target state by mimicking the complex paradigm of physiology, non-Ph.I.M. MOA can be described as “physiological.” This framing implies the need for evidence strategies that go beyond reductionist, single-constituent approaches. Accordingly, systems-level methods, including “omics” supported by bioinformatic analysis, have been proposed to capture matrix-level effects in relation to relevant pathophysiological features (disease “hallmarks”) and document the modulation of perturbed functions toward physiological conditions. Thus, “omics” approaches can help distinguish interaction patterns, showing that natural materials modulate multiple coordinated pathophysiological hallmarks, while pharmacological agents typically produce changes in a (potentially broad) targeted set. Above all, pharmacological agents allow causal traceability of the effects to the primary substance–target interaction. The “physiological” mode of action is thus specifically linked to natural materials, provided that their “naturalness” is appropriately defined and controlled (Mercati and Lucci, 2025b; Mercati and Lucci, 2025c).

## 6 Regulatory implications of the matrix effect of natural materials within Regulation 2017/745

The impossibility of representing the emergent properties of natural materials, such as vegetal matrices, through strict chemical features has implications for the General Safety and Performance Requirements (GSPRs), classification rules, and evaluation procedures that are relevant to medical devices made of natural materials (MDMNMs). Manufacturers are responsible for identifying the applicable GSPRs and the appropriate classification rule(s) to enable an accurate conformity assessment of the device; requirements and procedures specifically addressing substances/MDMS thus seem poorly suited to natural matrices. GSPRs 12.2, 23.2(r), 23.4(t), Rule 21 and the procedure in Annex IX relating to MDMS illustrate this potential mismatch. Treating natural materials as “a substance or a combination of substances” may lead to the inappropriate assessment of product characteristics, such as by accepting batches that do not deliver the desired biological output, discarding those that do, or failing to adequately evaluate network physiological MOAs.

This does not relax safety and quality expectations; rather, it implies that compliance with applicable GSPRs should be demonstrated through methods that are fit for purpose for the material and its intended action, consistent with MDR’s risk-based New Approach. Instead, a clear differentiation between natural-material devices and MDMS supports proportionate conformity assessment and avoids importing requirements designed for chemically definable substances into contexts where they may not be scientifically appropriate.

## 7 Discussion

The MDR, as “New Approach” legislation, requires and allows manufacturers to demonstrate compliance with the applicable GSPRs in a manner that is fit for purpose for the specific product. This flexibility is particularly valuable in a rapidly evolving context. The analysis above suggests that natural materials, such as vegetal matrices, cannot be adequately represented as well-defined substances; instead, they often require assessment approaches capable of capturing network interactions among constituents and with the human body. This differs from reductionist approaches that link effect primarily to defined molecular constituents. In natural matrices, supramolecular and functional interactions, together with redundancy among constituents, contribute to functional resilience, such that the relevant properties of the material are not fully described by any one or more constituents considered in isolation.

From a regulatory point of view, this supports a careful interpretation of the term “origin” within the MP definition of “substance”, defined by Directive 2001/83 as “any matter independently of its origin.” Consistent with the regulatory interpretation of “substance” analyzed above, “origin” under this directive, can be understood as indicating the source material from which the substance is derived (e.g., vegetal origin). If “substances” are operationalized as single, well-defined molecules, then a “substance of natural origin” is most coherently interpreted as a purified derivative obtained from natural starting materials (or presented as such), rather than the complex natural matrix itself. In this context, terms such as “complex substance,” “complex natural substance,” or “natural substance” are best interpreted as “substances of natural origin” when the product is presented and assessed as a substance, or as a material when assessed with a non-reductionist approach. Now that non-reductionist methods are emerging, it is possible to consider natural materials as a distinct category from substances, with distinct evidence generation and assessment, with no need to stretch experimental approaches and regulatory frameworks from substance settings.

**FIGURE 2:**
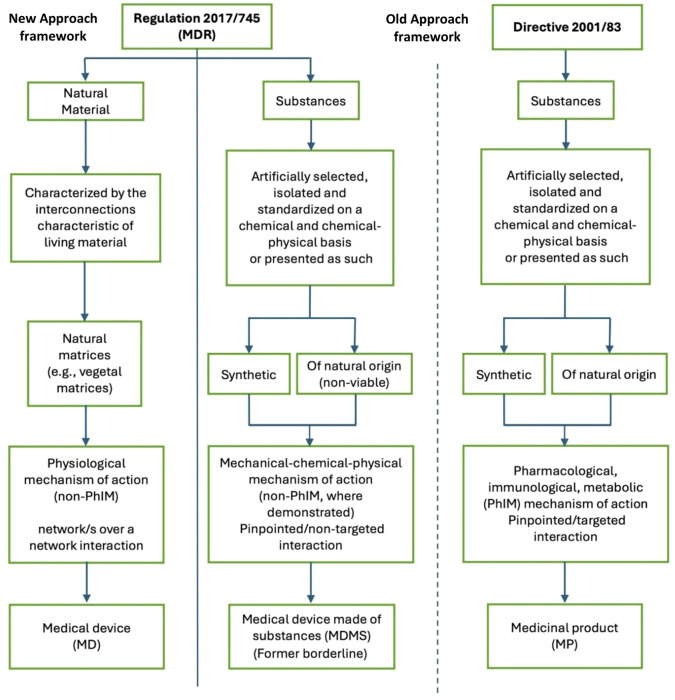
Regulatory pathway of a medical device, a medical device made of substances (also called “substance-based medical device”), and a medicinal product, taking into account the main characteristics of each as identified by the certification/authorization requirements. The fundamental difference is whether the product is made of substances (either synthetic or of natural origin) or is constituted of natural material/s. The other differences, including the mechanism of action, are derived from this. Medical devices made of substances and medicinal products share the fundamental characteristic of being made from substances. Natural materials cannot be described as “substances or combinations of substances”, and are, therefore, very different from both medical devices made of substances and medicinal products. MD, medical device; MDMS, medical device made of substances; MDR, Regulation 2017/745 Medical Device Regulation; MP, medicinal product; PhIM, pharmacological, immunological, metabolic. (modified from Marletta, 2024).

This reading is also compatible with Whereas 59, which highlights that substances entail specific risks when introduced into the human body. It is likely, in fact, that isolated substances (whether synthetic or purified), exogenous to the multiple interactions which govern the constituents of natural materials, expose patients to risks which need to be assessed as indicated by whereas 59 (ADME in particular). On the other hand, natural materials showing network interactions and the coordinated modulation of physiological processes require a different evidentiary approach. Products characterized by a physiological mode of action—that is, the modulation of perturbed functions toward physiological conditions through natural whole-matrix properties—can be more appropriately assessed under general MDR requirements, classification rules, and conformity assessment procedures rather than under MDMS-specific provisions (Figure 2). Reducing complex materials to single constituents is likely to limit the development of innovative products (Thomford et al., 2018). Notably, innovative botanical drugs remain relatively few, and several have involved substantial refinement to obtain selected constituents (e.g., flavonoids, catechins, triterpenes, or proteins) expressed in weight-based specifications (FDA, 2026b). Such approaches may not be appropriate for vegetal matrices which have performance that depends on the complexity of the matrix.

The implications of this evolving state of the art in product development remain to be fully defined. MDR places strong emphasis on clinical evidence, both pre- and post-market. For devices intended to modify a “state” of the patient, appropriate clinical study designs, endpoint selection, and assessment methods are likely to be needed to document relevant physiological effects. Physiological effects in non-therapeutic settings are already well established for complex food matrices under Regulation (EC) No 1924/2006 (EC, 2006). Through the definition of a medical device, which now makes reference to the modification of a physiological or pathological state and to information derived from specimens of the human body, it seems that MDR hopes for analogous effects to be brought within therapeutics and highlights the potential relevance of evidence derived from human specimens, including, where appropriate, “omics”-based approaches. Thus, emergent properties arising from the “native intelligence” of vegetal matrices—understood as functional behavior arising from native self-assembling properties, can inform each phase of product design and development, including clinical investigation.

Provided that quality is ensured throughout the life cycle of the device in accordance with standards such as ISO 13485, products developed within evidence-based “New Approach” regulatory frameworks can offer benefits in areas of unmet medical need, including degenerative diseases and oncology. Therefore, medical devices made of natural materials, when they meet the medical device definition in terms of nature (material), purpose (modification of a state), and non-Ph.I.M. (physiological) mode of action, can represent a meaningful innovative therapeutic approach.

Overall, this leads to a strategic choice: whether to explicitly recognize the difference between natural materials and substances and to develop natural-material devices within general MDR requirements or to treat natural materials as “substances” within reductionist, constituent-based paradigms. The former supports proportionate conformity assessment aligned with the scientific characteristics of natural matrices; the latter risks importing requirements designed for chemically definable substances into contexts where they are less appropriate, with potential consequences for innovation and access. On this basis, making full use of the MDR’s flexibility appears to be the preferable approach.
